# Supplementary material for: Development of a computer-aided design software for the quantitative evaluation of aesthetic damage
Source: PLoS One. 2019 Dec 18;14(12):e0226322. doi: 10.1371/journal.pone.0226322 (PMC6919621; doi:10.1371/journal.pone.0226322)
Supplement: S1 Code site — 1. (ZIP) [file pone.0226322.s001.zip › [EESC_jr][Projeto]Relatorio_07_21/reports/templates_zip/gentelella-master/production/project_detail.html]

Gentellela Alela! | 


Gentellela Alela!

Welcome,

## John Doe

  


### General

- Home 
  - Dashboard
  - Dashboard2
  - Dashboard3
- Forms 
  - General Form
  - Advanced Components
  - Form Validation
  - Form Wizard
  - Form Upload
  - Form Buttons
- UI Elements 
  - General Elements
  - Media Gallery
  - Typography
  - Icons
  - Glyphicons
  - Widgets
  - Invoice
  - Inbox
  - Calendar
- Tables 
  - Tables
  - Table Dynamic
- Data Presentation 
  - Chart JS
  - Chart JS2
  - Moris JS
  - ECharts
  - Other Charts
- Layouts 
  - Fixed Sidebar
  - Fixed Footer

### Live On

- Additional Pages 
  - E-commerce
  - Projects
  - Project Detail
  - Contacts
  - Profile
- Extras 
  - 403 Error
  - 404 Error
  - 500 Error
  - Plain Page
  - Login Page
  - Pricing Tables
- Multilevel Menu 
  - Level One- Level One
      - Level Two
      - Level Two
      - Level Two
    - Level One
- Landing Page Coming Soon

- John Doe
  - Profile
  - 50%
    Settings
  - Help
  - Log Out
- 6
  - John Smith
    3 mins ago

    Film festivals used to be do-or-die moments for movie makers. They were where...
  - John Smith
    3 mins ago

    Film festivals used to be do-or-die moments for movie makers. They were where...
  - John Smith
    3 mins ago

    Film festivals used to be do-or-die moments for movie makers. They were where...
  - John Smith
    3 mins ago

    Film festivals used to be do-or-die moments for movie makers. They were where...
  - **See All Alerts**


### Project Detail design

Go!

## New Partner Contracts Consultancy

- - Settings 1
  - Settings 2

- Estimated budget 
   2300
- Total amount spent 
   2000
- Estimated project duration 
   20

  

#### Recent Activity

- ### 24

  May

  #### Desmond Davison

  > Raw denim you probably haven't heard of them jean shorts Austin. Nesciunt tofu stumptown aliqua butcher retro keffiyeh dreamcatcher synth.

    

  User Acceptance Test.doc
- ### 21

  May

  #### Brian Michaels

  > Raw denim you probably haven't heard of them jean shorts Austin. Nesciunt tofu stumptown aliqua butcher retro keffiyeh dreamcatcher synth.

    

  Download
- ### 24

  May

  #### Desmond Davison

  > Raw denim you probably haven't heard of them jean shorts Austin. Nesciunt tofu stumptown aliqua butcher retro keffiyeh dreamcatcher synth.

    

  User Acceptance Test.doc

## Project Description

### Gentelella

Raw denim you probably haven't heard of them jean shorts Austin. Nesciunt tofu stumptown aliqua butcher retro keffiyeh dreamcatcher synth. Cosby sweater eu banh mi, qui irure terr.

  

Client Company

Deveint Inc

Project Leader

Tony Chicken

  

##### Project files

- Functional-requirements.docx
- UAT.pdf
- Email-from-flatbal.mln
- Logo.png
- Contract-10\_12\_2014.docx

  

Add files
Report contact


Gentelella - Bootstrap Admin Template by Colorlib
